# A Local TR-MUSIC Algorithm for Damage Imaging of Aircraft Structures

**DOI:** 10.3390/s21103334

**Published:** 2021-05-11

**Authors:** Shilei Fan, Aijia Zhang, Hu Sun, Fenglin Yun

**Affiliations:** 1 College of Aerospace Engineering, Nanjing University of Aeronautics & Astronautics, Nanjing 210016, China; fanshilei@comac.cc; 2 COMAC Shanghai Aircraft Design & Research Institute, Shanghai 201210, China; 3 School of Aerospace Engineering, Xiamen University, Xiamen 361005, China; aijia@stu.xmu.edu.cn (A.Z.); sunhu@xmu.edu.cn (H.S.)

**Keywords:** structural health monitoring, Lamb wave, damage imaging, local TR-MUSIC algorithm, superresolution

## Abstract

Lamb wave-based damage imaging is a promising technique for aircraft structural health monitoring, as enhancing the resolution of damage detection is a persistent challenge. In this paper, a damage imaging technique based on the Time Reversal-MUltiple SIgnal Classification (TR-MUSIC) algorithm is developed to detect damage in plate-type structures. In the TR-MUSIC algorithm, a transfer matrix is first established by exciting and sensing signals. A TR operator is constructed for eigenvalue decomposition to divide the data space into signal and noise subspaces. The structural space spectrum of the algorithm is calculated based on the orthogonality of the two subspaces. A local TR-MUSIC algorithm is proposed to enhance the image quality of multiple damages by using a moving time window to establish the local space spectrum at different times or different distances. The multidamage detection capability of the proposed enhanced TR-MUSIC algorithm is verified by simulations and experiments. The results reveal that the local TR-MUSIC algorithm can not only effectively detect multiple damages in plate-type structures with good image quality but also has a superresolution ability for detecting damage with distances smaller than half the wavelength.

## 1. Introduction

Structural health monitoring (SHM) has been considered a revolutionary technology for the operation and maintenance of aircraft structures [1]. At present, some SHM technologies, such as acoustic emission [2,3], eddy current [4,5,6], Lamb wave [7,8], impedance [9,10], vibration [11,12], and comparative vacuum [13] monitoring, have received greatly focused research and development for damage detection in aircraft structures. Lamb wave-based damage imaging, which is an active detection technology suitable for large-scale structures, is a promising technology for the health monitoring of aircraft plate structures [14,15].

However, due to the multiple modes, wave dispersion and boundary scattering of Lamb waves, the acquired Lamb wave signal is often difficult to interpret and needs to be further analyzed. With the help of advanced signal and information processing algorithms, Lamb wave damage imaging technology, which can intuitively provide the location and size of the damage, has become a topic of interest in SHM technology. Existing Lamb wave damage imaging technologies, such as probabilistic reconstruction algorithms [16,17], phased arrays [18,19], tomography [20,21] and delay-and-sum [22,23] methods, can realize damage location and even quantification in plate-type structures. However, due to the limitations of wave diffraction, existing damage imaging algorithms have poor resolution and difficulty distinguishing two neighboring damage within the Rayleigh limit. The deficiency of damage monitoring capability makes it impossible to acquire sufficient information to predict the residual life of structures, which limits the further development of Lamb wave-based SHM technology.

Multiple signal classification (MUSIC) is a typical method used to address the Rayleigh criterion. Schmidt et al. [24] proposed the MUSIC algorithm based on eigenvalue decomposition and subspace theory. The algorithm offers high estimation resolution and stability for estimating the direction of a target and has been widely used in electromagnetic waves, acoustic waves and other fields. For wave-based damage identification, each damage can be regarded as a secondary wave source according to the Huygens principle, which means that the MUSIC algorithm can be used to estimate the damage location. Engholm et al. [25] used the MUSIC algorithm to estimate the direction of the damage source but did not provide the damage distance. Ambrozinski et al. [26] applied the MUSIC algorithm for the damage location of an aluminum plate. Zhong et al. [27,28,29] developed a series of improved MUSIC algorithm to detect impact sources under varying temperature, vibration and deformation conditions. Yuan et al. [19,30,31] made use of the MUSIC algorithm for damage location estimation of aircraft structures.

Time reversal multiple signal classification (TR-MUSIC), which combines the adaptive focusing of the time reversal algorithm and the superresolution of direction estimation of the MUSIC algorithm, has also been used in ultrasonic nondestructive testing. Devaney et al. [32] first proposed TR-MUSIC and verified its superresolution ability in identifying two parallel copper wires with a short distance in water by numerical simulations. Simonetti [33] used ultrasonic TR-MUSIC to create defect images for the first time and successfully distinguished two holes with half wavelengths in a steel block, which broke through the limitation of acoustic diffraction and realized the ultrasonic superresolution imaging of solid media. Fan et al. [34] verified the excellent performance of TR-MUSIC in superresolution damage imaging by comparing TR-MUSIC with the full focus method (TFM). Several studies assessed TR-MUSIC for the damage detection of plate-type structures by using ultrasonic Lamb waves. He and Yuan [35] imaged multiple damages over a long distance in an aluminum plate by using a piezoelectric array and the DORT-MUSIC algorithm. However, their study did not discuss the superresolution capability of detecting multiple damages. In addition, two other problems exist in multidamage detection. One is image interference caused by pixel values of different orders of magnitude at different damage locations, which results from different damage sizes and different distances from the damage to the sensing array. Another is that when the amount of damage is large and even larger than the sensor numbers, the TR-MUSIC algorithm is prone to generating an incorrect damage image.

In this paper, an enhanced TR-MUSIC algorithm is proposed to study the capability of detecting multiple damages in plate-type structures. The superresolution ability to detect multiple damages, whose distances are smaller than a half-wavelength, is discussed. In addition, the enhanced algorithm can markedly improve the quality of multidamage images.

The structure of this paper is established as follows. In Section 2, the process and formulations of TR-MUSIC are introduced. In Section 3, the ability of the local TR-MUSIC algorithm is verified for multidamage detection by using the signals from the finite element model. The algorithm is further verified by experiments in Section 4. Finally, conclusions are drawn in Section 5.

## 2. Local TR-MUSIC Algorithm

The basic process and theoretical formulations have been introduced in the references [29,30,31,32]. In this paper, the formulations of the TR-MUSIC algorithm based on a linear piezoelectric sensor array and Lamb waves can be written as follows.

### 2.1. Transfer Matrix

**Figure 1:**
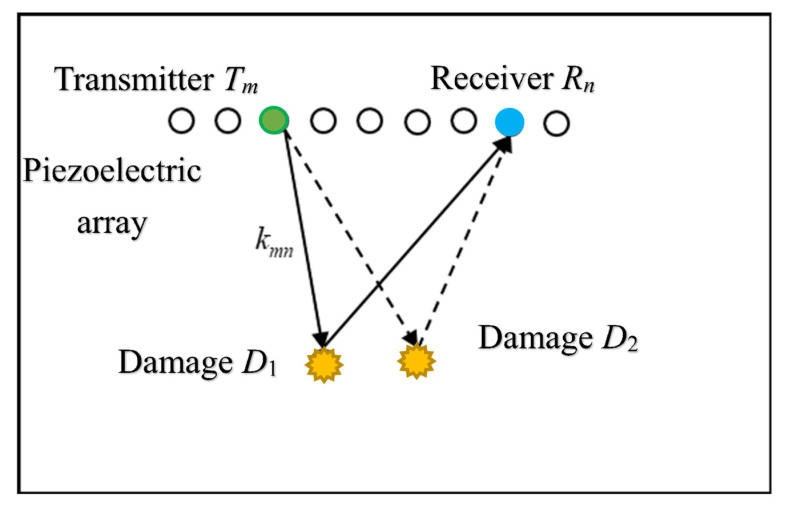
Piezoelectric array and transfer matrix.

Figure 1 shows an operational diagram of a piezoelectric sensor array. A piezoelectric array containing *N* elements is bonded to the surface of a plate, and piezoelectric elements are used not only as transmitters but also as receivers. Each element in the piezoelectric array is excited in turn, and all the array elements receive response signals. Assume that $T_{m}(m=1,2,\cdots N)$ represents the transmitter location, $R_{n}(n=1,2,\cdots ,N)$ represents the receiver location, and $D_{q},\;q=1,2\cdots ,Q$ represents the damage location.

The *m*th (1≤m≤N) piezoelectric element in the piezoelectric array is used as a transmitter to excite the Lamb wave propagating in the structure. The wave at location $r_{s}$ away from the transmitter can be expressed as

(1) $$y_{sm}(t)=g(r_{s},T_{m},t)⊗x(t)$$

where $g(r_{s},T_{m},t)$ is Green’s function, which represents the process of the Lamb wave propagating from $T_{m}$ to $r_{s}$. In the frequency domain, Equation (1) can be written as

(2) $$Y_{sm}(\omega )=G(r_{s},T_{m},\omega )X(\omega )$$

where X(ω), $Y_{sm}(\omega )$ and $G(r_{s},T_{m},\omega )$ are Fourier transforms of x(t), $y_{sm}(t)$ and $g(r_{s},T_{m},t)$, respectively. The frequency symbol ω will be omitted in the following mathematical expression.

After scattering by the damage at $r_{s}$, the Lamb wave is acquired by the *n*th receiver $R_{n}$ as follows:

(3) $$Y_{nsm}=G(R_{n},r_{s})O(r_{s})Y_{sm}=G(R_{n},r_{s})O(r_{s})G(r_{s},T_{m})X$$

where $O(r_{s})$ is the scattering coefficient of the damage.

For point-type damage, the influence of different damage can be neglected, and then the scattering coefficient of the structure can be expressed as follows:

(4) $$O(r)=\sum_{q=1}^{Q}\sigma_{q}(r_{q})\delta (r-r_{q})$$

where $\sigma_{q}(r_{q})$ is the scattering coefficient of the damage at $r_{q}$ and $\delta (r-r_{q})$ is the Dirac function.

Substituting Equation (4) into Equation (3), the signal that is sensed by the *n*th receiver and transmitted by the *m*th transmitter can be written as

(5) $$Y_{nsm}=\sum_{q=1}^{Q}G(R_{n},r_{q})\sigma_{q}G(r_{q},T_{m})X$$

Then, the transfer matrix element can be defined as

(6) $$K_{nm}=\sum_{q=1}^{Q}G(R_{n},r_{q})\sigma_{q}G(r_{q},T_{m})$$

where the subscript ‘*nm*’ indicates that the *m*th sensor is used as the transmitter and the *n*th sensor is used as the receiver. Therefore, the transfer matrix can be defined as

(7) $$\bar{K}=\bar{G}(R,r)\bar{O}(r){\bar{G}}^{T}(r,T)$$

where R, r and T represent the locations of receivers, damage and transmitters, respectively. $\bar{G}(R,r)$ and ${\bar{G}}^{T}(r,T)$ are Green’s function matrices, which are associated sets of Green’s functions $G(R_{n},r_{q})$ and $G(r_{q},T_{m})$, respectively.

### 2.2. Time-Reversal Operation

The TR process can be conducted from the transfer function matrix.

If ${\bar{X}}^{\left(0\right)}$ and ${\bar{Y}}^{\left(0\right)}$ are the input and output matrices, respectively, the following equation can be obtained:

(8) $${\bar{Y}}^{\left(0\right)}=\bar{K}{\bar{X}}^{\left(0\right)}$$

The time reversal of the output signal is equal to the phase conjugation of the frequency-domain signal, and it yields

(9) $${\bar{X}}^{\left(1\right)}={\left({\bar{Y}}^{\left(0\right)}\right)}^{*}={\bar{K}}^{*}{\left({\bar{X}}^{\left(0\right)}\right)}^{*}$$

where the symbol ‘*’ indicates the conjugate operation on the matrix and the superscript ‘1’ indicates the first time-reversal operation. ${\bar{X}}^{\left(1\right)}$ is considered to be the new input signal and is emitted, and the new output matrix can be written as

(10) $${\bar{Y}}^{\left(1\right)}=\bar{K}{\bar{X}}^{\left(1\right)}=\bar{K}{\bar{K}}^{*}{\left({\bar{X}}^{\left(0\right)}\right)}^{*}$$

${\bar{Y}}^{\left(1\right)}$ is reversed again as the new input matrix

(11) $${\bar{X}}^{\left(2\right)}={\left({\bar{Y}}^{\left(1\right)}\right)}^{*}={\bar{K}}^{*}\bar{K}{\bar{X}}^{\left(0\right)}$$

After the 2*n* iterations, the input matrix of the system is written as

(12) $${\bar{X}}^{\left(2n\right)}={\left({\bar{K}}^{*}\bar{K}\right)}^{n}{\bar{X}}^{(0)}$$

After the 2*n* + 1 iterations, the new input matrix ${\bar{X}}^{\left(2n+1\right)}$ is

(13) $${\bar{X}}^{\left(2n+1\right)}={\bar{K}}^{*}{\left(\bar{K}{\bar{K}}^{*}\right)}^{n}{\left({\bar{X}}^{\left(0\right)}\right)}^{*}$$

From Equations (12) and (13), two kinds of time reversal operators are defined as

(14) $${\bar{T}}_{1}={\bar{K}}^{*}\bar{K},\;{\bar{T}}_{2}=\bar{K}{\bar{K}}^{*}$$

where ${\bar{T}}_{1}$ and ${\bar{T}}_{2}$ are the time reversal operators for the transmitting and receiving modes, respectively.

If the transfer matrix is a complex symmetric matrix, the following feature of the TR operation can be obtained:

(15) $${\bar{T}}^{H}={\left({\bar{K}}^{*}\bar{K}\right)}^{H}={\bar{K}}^{H}{\left({\bar{K}}^{*}\right)}^{H}={\bar{K}}^{*}\bar{K}=\bar{T}$$

where the symbol ‘*H*’ denotes the conjugate transposition operation. From Equation (15), the conjugate transposition of the TR operator is equal to itself, that is, $\bar{T}$ is the Hermite matrix. There are two features for the Hermite matrix $\bar{T}$: (a) all eigenvalues of time reversal operators are real; (b) eigenvectors of time reversal operators are orthogonal to each other.

### 2.3. Singular Value Decompositions

The singular value decompositions of the transfer matrix and its conjugate transposition can be expressed as

(16) $$\bar{K}=\bar{U}\bar{\Lambda }{\bar{V}}^{H},\;{\bar{K}}^{H}=\bar{V}{\bar{\Lambda }}^{H}{\bar{U}}^{H}$$

(17) $$\Lambda =\left[\begin{array}{cccc} \sigma_{1} & & & \\ & \sigma_{2} & & \\ & & \ddots & \\ & & & \sigma_{N} \end{array}\right]$$

(18) $$\bar{K}=\bar{U}\bar{\Lambda }{\bar{V}}^{H},\;{\bar{K}}^{H}=\bar{V}{\bar{\Lambda }}^{H}{\bar{U}}^{H}$$

(19) $$\bar{V}=[{\bar{V}}_{signal}|{\bar{V}}_{noise}]=[{\bar{\nu }}_{1},{\;\bar{\nu }}_{2},\cdots ,{\;\bar{\nu }}_{Q}|{\;\bar{\nu }}_{Q+1},\cdots ,{\bar{\nu }}_{N}]$$

where $\sigma_{i}$ is defined as the singular value of the transfer matrix, while ${\bar{\mu }}_{i}$ and ${\bar{\nu }}_{i}$ are the left and right singular vectors related to $\sigma_{i}$, respectively.

The eigenvalue decompositions of ${\bar{T}}_{1}$ and ${\bar{T}}_{2}$ can be expressed as follows:

(20) $${\bar{T}}_{1}={\bar{K}}^{*}\bar{K}={\bar{K}}^{H}\bar{K}=\bar{V}{\bar{\Lambda }}^{2}{\bar{V}}^{H}$$

(21) $${\bar{T}}_{2}=\bar{K}{\bar{K}}^{*}=\bar{K}{\bar{K}}^{H}=\bar{U}{\bar{\Lambda }}^{2}{\bar{U}}^{H}$$

The eigenvalue decomposition of the TR operator is used to divide the whole data space into a signal subspace and noise subspace according to the distribution of eigenvalues. Under the assumption of ideal point damage, there is a one-to-one mapping relationship between each damage on the structure and the eigenvector corresponding to each nonzero eigenvalue of the TR operator. Therefore, the vector space composed of eigenvectors corresponding to nonzero eigenvalues is usually regarded as the signal subspace, while the vector space composed of eigenvectors corresponding to zero eigenvalues is regarded as the noise subspace.

### 2.4. TR-MUSIC Imaging Algorithm

The basis of the signal subspace is obtained by eigenvalue decomposition of the TR operator. Under the Born approximation, the signal subspace can also be calculated by Green’s function. The signal subspace and noise subspace are orthogonal to each other so that the inner product of the noise subspace and the Green function vector at the real damage site should be equal to 0. The imaging function of *TR-MUSIC* is defined as

(22) $$I_{TR-MUSIC}^{q}(r_{s})=\frac{1}{\sum_{i=Q+1}^{N}{\left|\langle {\bar{\mu }}_{i},\bar{G}(R_{n},r_{s})\rangle \right|}^{2}}$$

where Green’s function at each discrete point of the structure is used to calculate the inner product with the noise subspace to construct the spatial spectrum of the TR-MUSIC algorithm. The peak of the spatial spectrum is the location of the real damage.

### 2.5. Local TR-MUSIC Imaging Algorithm

TR-MUSIC has a poor capability of detecting multiple damages, especially when the element number of the sensor array is small. Therefore, to solve the above problem, an enhanced monitoring strategy based on the local TR-MUSIC algorithm has been developed.

The process of the local TR-MUSIC algorithm is similar to the short-time Fourier transform. First, a Hanning window moving along the time axis is used to separate the original scattered signal $y_{sm}(t)$ into a set of short-segment signals:

(23) $$z_{i}(t)=y_{sm}(t)w(t-t_{i})\;0<i<I$$

where $w(t-t_{i})$ denotes the Hanning window function at the *i*th time point and *I* represents that the signal is separated into *I* segments. Different *Is* denote different step sizes in which the window moves, and the larger the value of *I* is, the smaller the step size of the window function is. The local segments of scatter signals $z_{i}(t)$ are used to calculate the local transfer matrix and perform the local eigenvalue decomposition. Thus, there exists a time-dependent eigenvalue function. Generally, it can be imagined that the signal segments that are scattered from the real damage will generate a local maximum point in the eigenvalue function. When the damages have different distances from the sensor array, the time of scattering signals caused by each damage is different, and the time of the local maximum point in the eigenvalue function is also different. This process can be expressed as

(24) $$\tilde{t}=\underset{i}{\arg\max}\mu \left(ℜ\left(\kappa \left(z_{i}(t)\right)\right)\right)$$

where κ(•) denotes the transfer function, which is the quotient of the scattering signal and incident signal in the frequency domain. ℜ(•) and μ(•) denote the process of calculating the TR operator and the eigenvalue, respectively, whose formulations can be seen in Equations (15) and (16). For different *i*, $\mu \left(ℜ\left(\kappa \left(z_{i}(t)\right)\right)\right)$ makes a time-dependent eigenvalue function. Thus, ‘argmax’ means looking for the time when the eigenvalue function takes the local maximum. $\tilde{t}$ is just a set of time points where local maximum values exist. Thus, for short-segment signals $z_{i}(t)$ at each time point in $\tilde{t}$, a damage image performed by Equation (22) can locate the real damage. The multidamage contour can be summed by the damage images at all time points in $\tilde{t}$, which is written by

(25) $${\tilde{I}}_{TR-MUSIC}^{q}(r_{s})=\sum_{\tilde{t}}\frac{1}{\sum_{i=Q+1}^{N}{\left|\langle \mu_{i}\left(ℜ\left(\kappa \left(z(\tilde{t})\right)\right)\right),\bar{G}(R_{n},r_{s})\rangle \right|}^{2}}$$

This can effectively avoid the occurrence of missing damage. In addition, since the scattered signals of different time periods are cut out for damage location processing from zero, this is equivalent to scanning the structure from near to far according to the distance from each structural point to the sensing array. At the same distance, the probability that the damage number exceeds the total number of sensing array elements is almost negligible. Therefore, this method can also solve the problem whereby the damage number identified by the TR-MUSIC algorithm cannot exceed the total number of elements in the sensing array.

## 3. Simulation Verification

**Figure 2:**
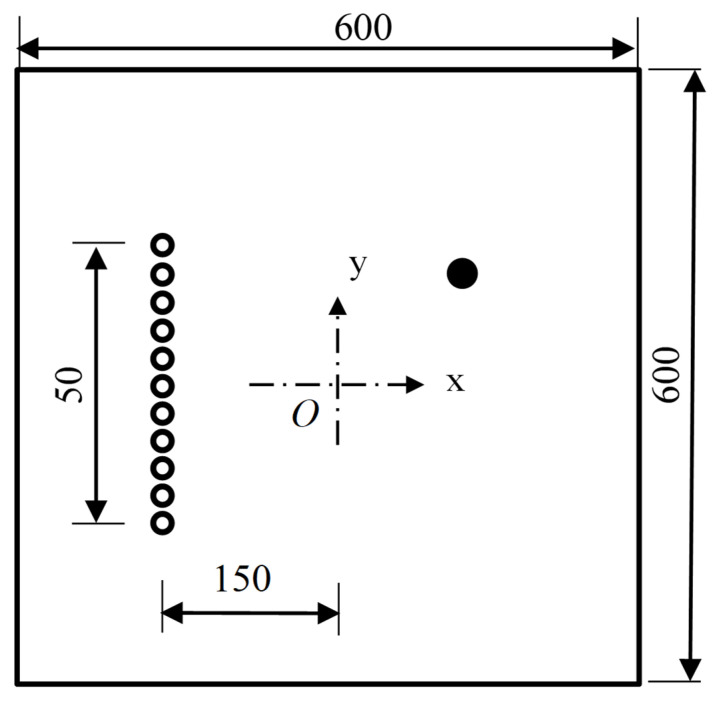
Schematic diagram of finite element model.

A finite element simulation based on the ABAQUS Standard is used to verify the effectiveness and feasibility of the developed damage imaging algorithm. In Figure 2, an aluminum plate with dimensions of 600 mm × 600 mm × 1 mm, an elastic modulus of 70 GPa, a Poisson’s ratio of 0.3, and a mass density of 2700 kg/m^3^ is considered. The boundary of the plate is fixed all around. The element type is C3D8R, and the element size is 1 mm. There are a total of 360,000 elements. The time interval is set as 0.1 μs, and the recording time is 0.5 ms. The exciting signal, with a five-peaked Hanning windowed sinusoidal tone burst and a center frequency of 50 kHz, is loaded along the in-plane direction at array nodes to model the piezoelectric sensor. Taking the center point as the coordinate origin, a linear sensor array with 11 array elements spaced at 5 mm lies at the left of and 150 mm away from the coordinate origin.

### 3.1. Single- and Multi-Damage Identification

In this section, two damage cases—a single damage and three damages—are considered. Based on the coordinates in Figure 2, a few damage locations are set casually, and detailed damage information is given in Table 1.

**Figure 3:**
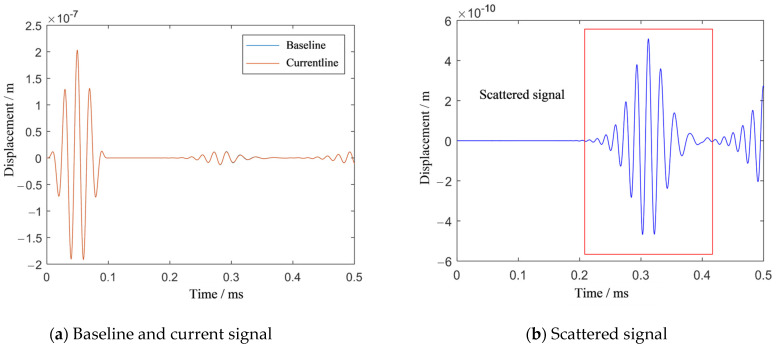
The effect of damage on the signals in Case I.

Figure 3a shows the baseline and current signals recorded by the 6th sensor. The signals before and after damage show almost no change. Then, the scattered signal, with a smaller magnitude than the incident wave in Figure 3a, is obtained by subtracting the baseline signal from the current signal, as shown in Figure 3b. The first wave packet in the signal is the scattering signal caused by damage, and the subsequent wave packet is the scattered signal from the boundary. The scattering signal caused by the damage is extracted to calculate the transfer matrix and the TR operator, and eigenvalue decomposition of the TR operator is performed.

**Figure 4:**
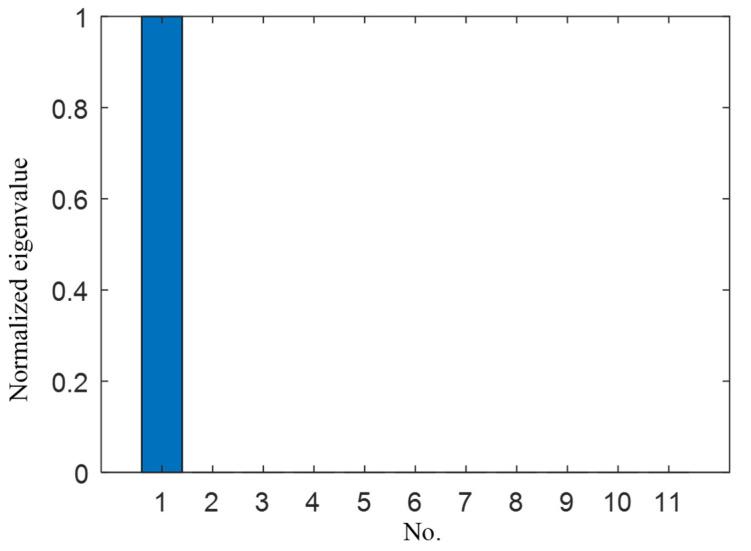
Normalized eigenvalue distribution of Case I.

**Figure 5:**
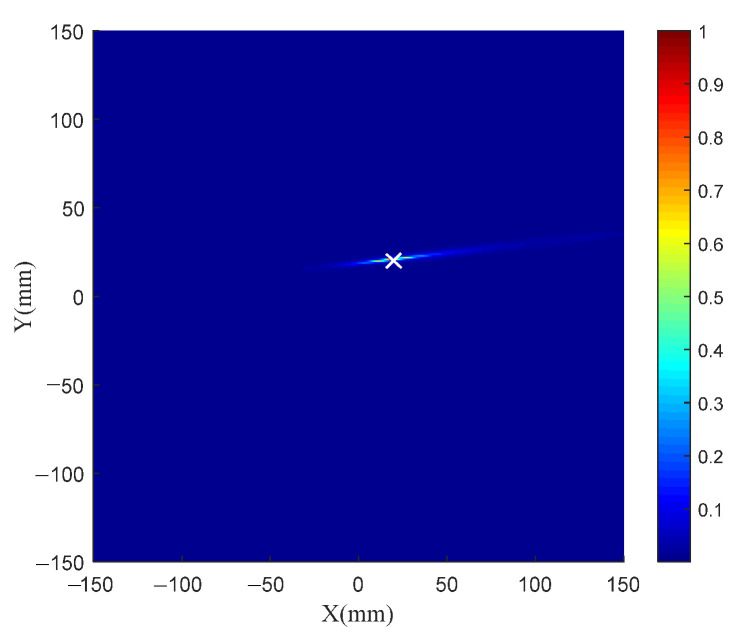
TR-MUSIC imaging results for Case I.

Figure 4 shows the normalized eigenvalue distribution of the TR operator. There is only one nonzero eigenvalue, and the other eigenvalues are all zeros. The signal subspace and the noise subspace can be obtained. The spatial spectrum of the TR-MUSIC algorithm is constructed to identify the damage. Figure 5 shows the imaging results of Case I in Table 1. The symbol ‘X’ indicates the location of the real damage in the structure. The peak-value location of the spatial spectrum coincides with the real damage location of the structure, which means that the TR-MUSIC algorithm successfully realizes the damage image of a single damage.

**Figure 6:**
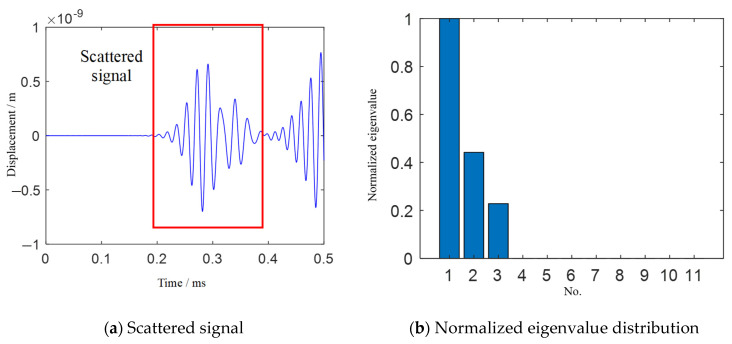
Scattered signal and eigenvalue distribution of Case II.

**Figure 7:**
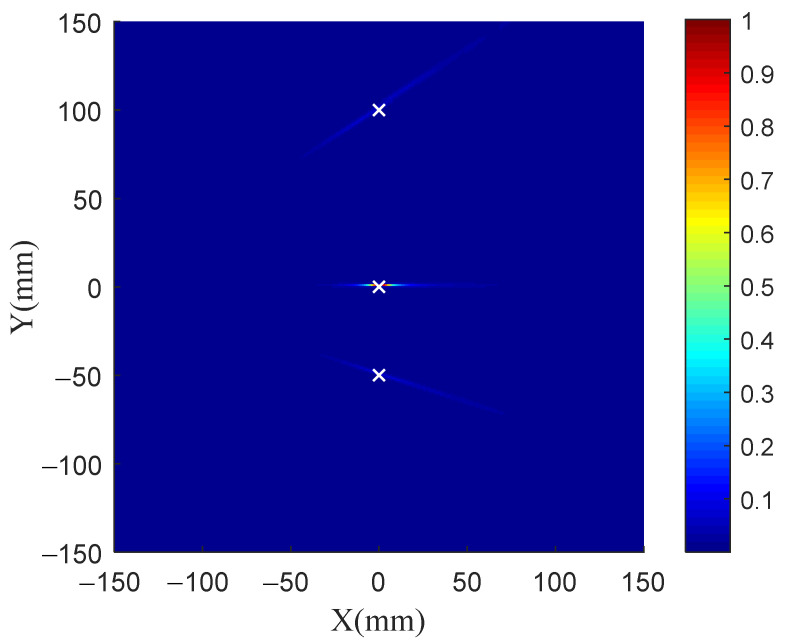
TR-MUSIC imaging results for Case II.

Figure 6 and Figure 7 show the scattered signals, eigenvalue distribution and damage imaging results of three damages. Multiple damage imaging is also successfully realized by TR-MUSIC.

### 3.2. Verification of Local TR-MUSIC Algorithm

The same model as shown in Figure 2 is considered. Three damages are set in the model. Damage sizes are all 4 mm × 4 mm × 1 mm. Damage coordinates are (−50 mm, −50 mm), (0 mm, 0 mm) and (50 mm, 50 mm).

**Figure 8:**
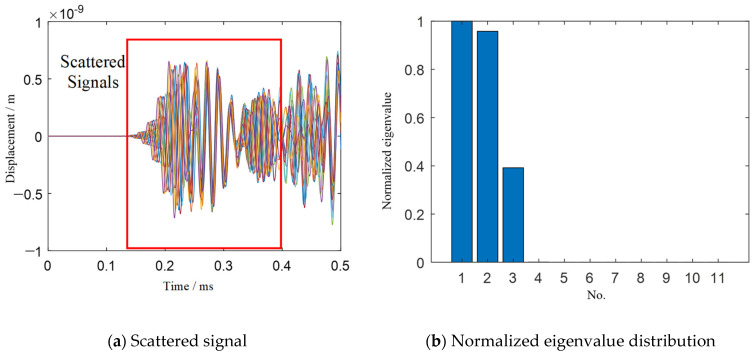
Scattered signals and eigenvalue distribution of three damage for TR-MUSIC.

Figure 8a shows the scattered signal received by all sensing elements. Figure 8b shows the normalized eigenvalue distribution. There are three nonzero eigenvalues, which are consistent with the number of damage in the structure. Therefore, the vector space composed of the eigenvectors corresponding to the three nonzero eigenvalues is taken as the signal subspace, while the vector space composed of the other eigenvectors corresponding to the zero eigenvalues is used as the noise subspace for damage identification.

**Figure 9:**
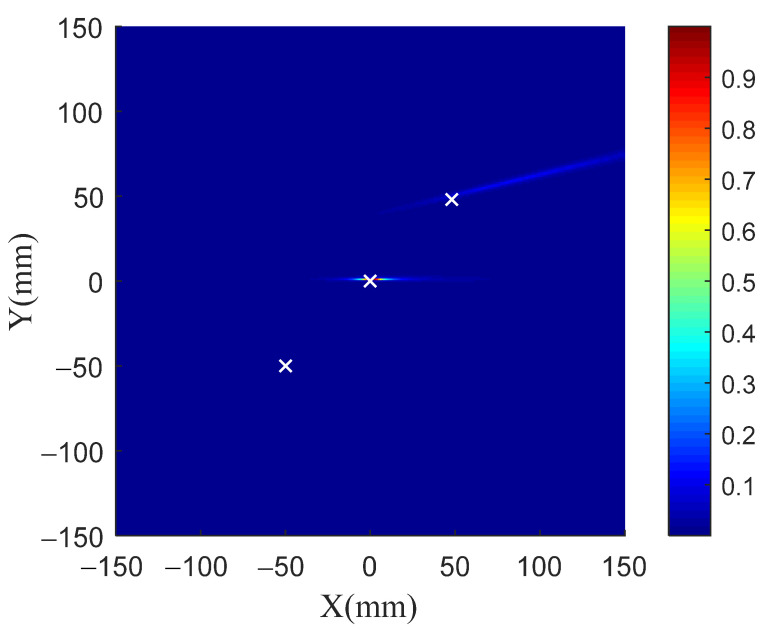
Imaging results of three damage for TR-MUSIC.

Figure 9 shows the imaging results of the TR-MUSIC algorithm for three damage. Comparisons between the identified damage locations, which are obtained from the local peak locations in the image, and the real damage locations can be seen in Table 2. Figure 9 and Table 2 show that only the damage at the location (0 mm, 0 mm) has a high imaging accuracy. For the damage at the location (50 mm, 50 mm), although the damage direction identification is correct, the identification error of radial distance is very large. Moreover, the damage at the location (−50 mm, −50 mm) is not even visible in the damage image.

**Figure 10:**
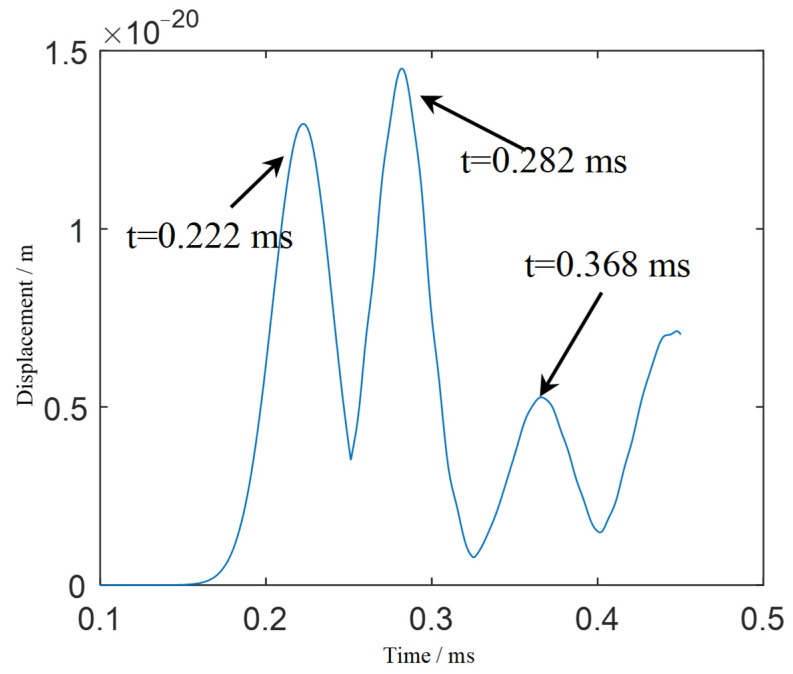
Time-dependent eigenvalue function calculated by local TR-MUSIC.

To solve this problem, the local TR-MUSIC algorithm is applied. The Hanning window length is one-fifth of the length of the exciting signal. Figure 10 shows the calculated time-dependent eigenvalue function. There are three extreme points in the function, corresponding to 0.222 ms, 0.282 ms and 0.368 ms. Therefore, the scattering signals at these three moments are imaged.

**Figure 11:**
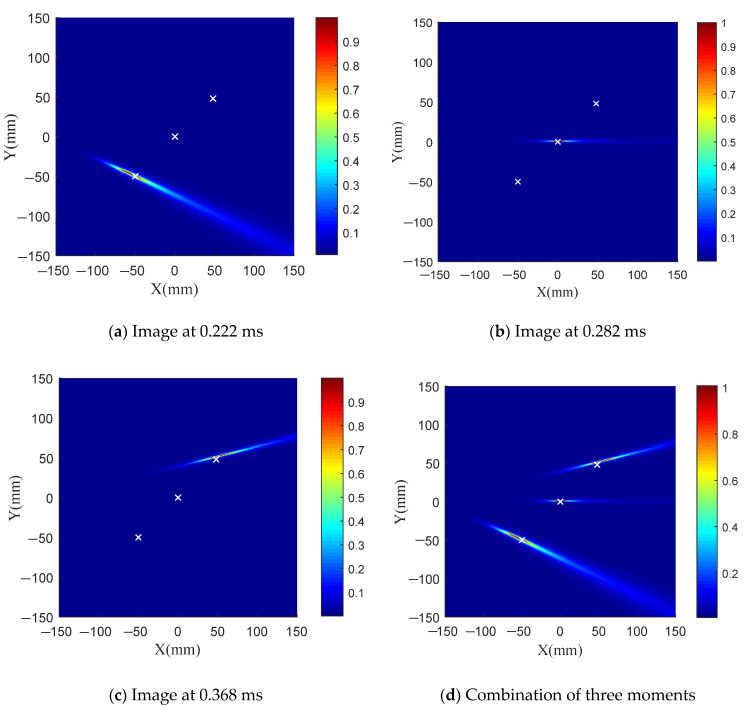
Damage imaging by using local TR-MUSIC algorithm.

Figure 11a–c show the imaging results of these three moments, and Figure 11d shows the combined imaging results. The local TR-MUSIC algorithm is capable of identifying multiple damages, and it can realize damage identification from near to far according to the distance from each structural point to the sensing array. Even when the damage number is larger than the array element number, the local TR-MUSIC can provide good damage identification ability.

### 3.3. Super-Resolution Imaging

In this section, the superresolution capability of the TR-MUSIC algorithm is verified. By dispersion analysis, the wavelength of the A0 wave at the frequency-thickness product of 50 kHz·mm is 13.7 mm. In the same model, as shown in Figure 2, two damage with a size of 4 mm × 4 mm × 1 mm are set at (0 mm, −3 mm) and (0 mm, 3 mm), respectively. Thus, the central distance between these two damages is 6 mm, and the shortest distance is only 2 mm.

**Figure 12:**
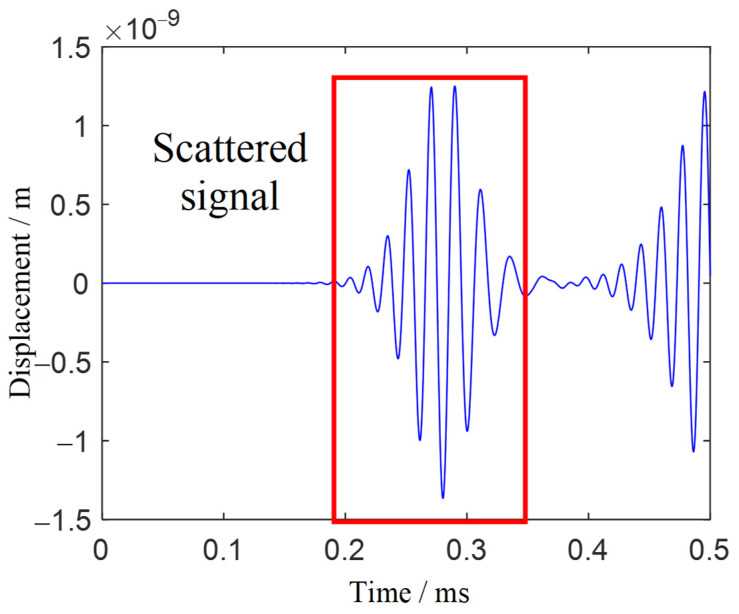
Scattered signal of two close damage.

**Figure 13:**
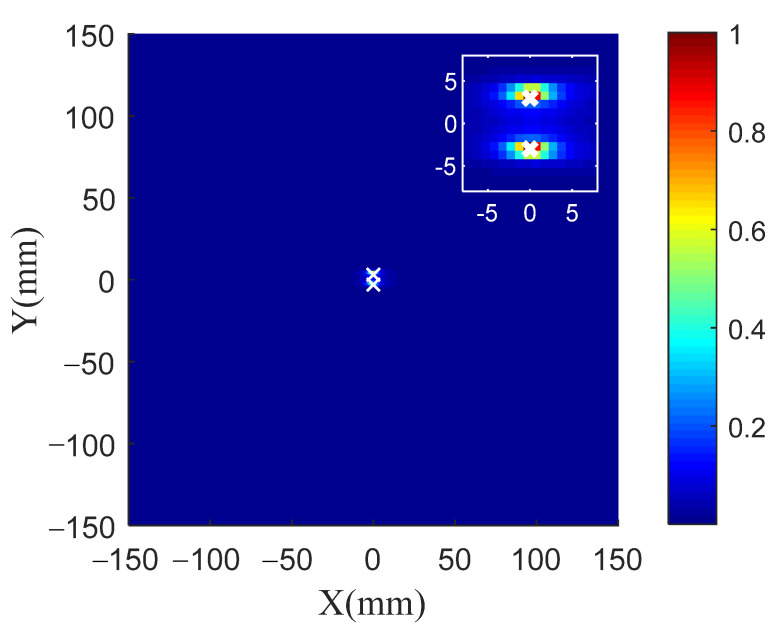
TR-MUSIC imaging of damage with distance less than half-wavelength.

Figure 12a shows the scattered signal. There is only one scattering wave packet in the signal, so it is impossible to distinguish the two damage types from the time domain signal. The scattered signal caused by the damage is substituted into the TR-MUSIC algorithm. The eigenvalues are shown in Table 3, and it can be seen that eigenvalues No. 1 and No. 2 are quite larger than the other eigenvalues. Therefore, the vector space composed of the eigenvectors corresponding to the first two eigenvalues is taken as the signal subspace, and the vector space composed of the eigenvectors corresponding to the other eigenvalues is taken as the noise subspace. Based on the orthogonality of the Green function and noise subspace, the spatial spectrum of the TR-MUSIC algorithm is constructed. Figure 13 shows the TR-MUSIC imaging results. It can be seen that the superresolution imaging of damage with a less than half-wavelength distance has been successfully realized.

### 3.4. Enhancing Radial Resolution Using Dual Arrays

**Figure 14:**
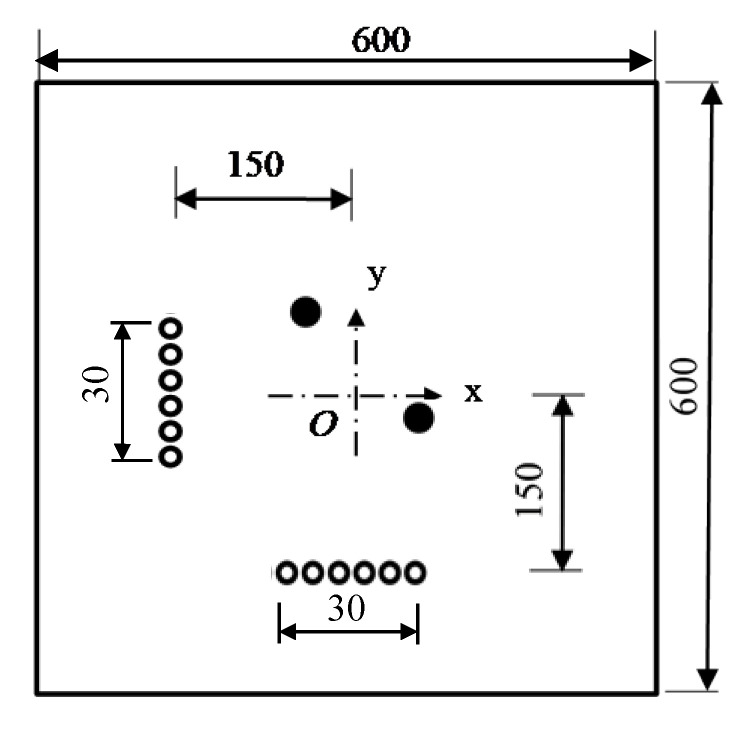
Simulation model containing dual arrays.

**Figure 15:**
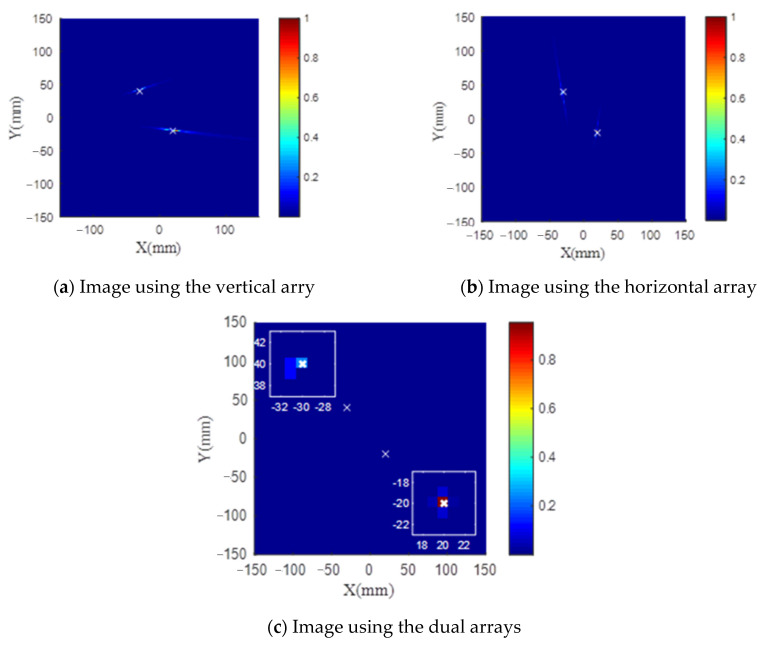
Comparisons of damage image between single and dual arrays.

It can be seen from the previous sections that the proposed TR-MUSIC algorithm has a good angle resolution for damage detection but a poor radial resolution. In order to eliminate this problem, dual arrays having an ‘L’ shape can be used for damage imaging. As shown in Figure 14, one horizontal and one vertical array containing 6 sensors are used to detect two damage in the aluminate panel. The same process is used for both horizontal and vertical arrays. Figure 15 gives comparisons of damage images between single array and dual arrays. It is obvious that although a single array has also a poor radial resolution, dual arrays have eliminated this illustration by a simple summation of two damage images.

## 4. Experimental Section

**Figure 16:**
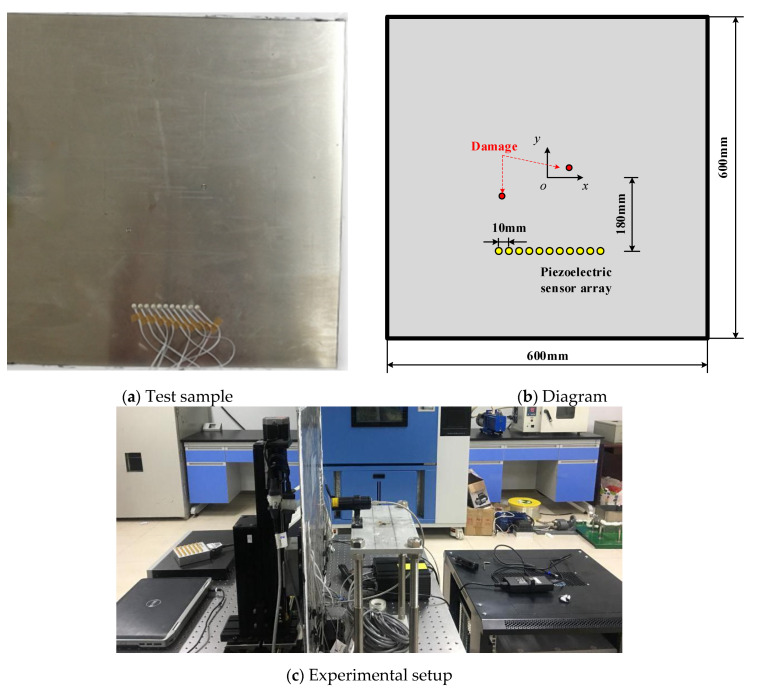
Test sample and experimental setup.

An experiment was conducted to verify the feasibility and effectiveness of the proposed algorithm. As shown in Figure 16, the test sample was an aluminum panel with dimensions of 600 mm × 600 mm × 2 mm. Eleven PZT sensors that formed a linear piezoelectric sensor array were bonded to the surface of the aluminum panel. The diameter of the sensor is 8 mm, and the thickness is 0.48 mm. The distance of the adjacent sensors is 10 mm. Taking the panel center as the coordinate origin, the distance between the coordinate origin and the linear array is 180 mm. As shown in Figure 16c, a single-transmitting-and-multiple-receiving guided wave monitoring system based on the piezoelectric sensors developed by Xue et al. [33] was used to acquire signals. A five-peaked Hanning windowed sinusoidal tone burst with a center frequency of 70 kHz was used as the exciting signal. Holes were drilled in the aluminum panel to model the real damage. Two damage cases, including one damage and two damage, were considered in the experiment.

**Figure 17:**
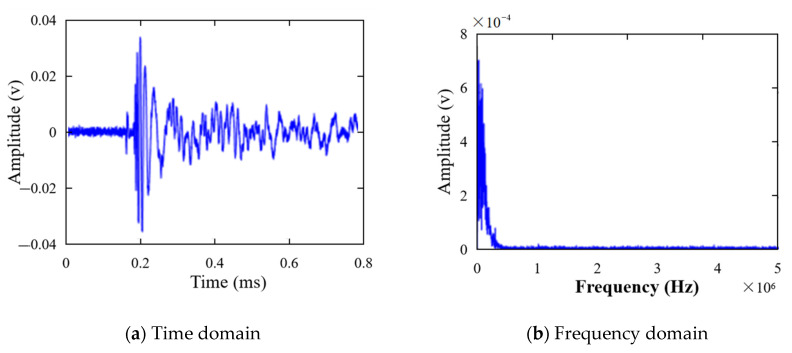
The original broadband signal excited by pulse laser.

**Figure 18:**
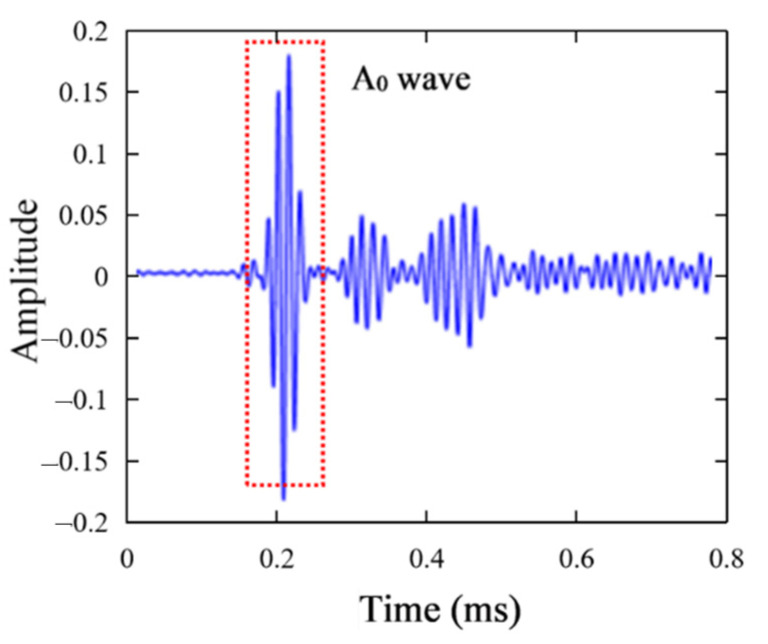
Narrowband signal extracted by wavelet transform.

To obtain accurate Green functions, a hybrid system combining a Nd:Yag pulse laser and a piezoelectric sensor was also developed, as shown in Figure 16c. The aluminum panel was fixed on an X-Y scanner to move to realize scanning of all the monitored area, while a Nd:Yag pulse laser was fixed on the optical platform to excite the Lamb wave propagating in the panel. Hence the plate can be monitored on a rectangular arrays for the positions of the laser source. The piezoelectric sensor array in Figure 16a was connected to an acquisition card to collect signals to calculate the green functions. Figure 17a,b shows a typical broadband signal acquired by the piezoelectric sensor when the pulse laser was used for excitation. To calculate the Green functions at a frequency of 70 kHz, the Gabor wavelet transform was used to extract the narrowband signal. As shown in Figure 18, the A0 wave was extracted to perform Fourier transform and calculate the Green function. For one exciting point of the pulse laser, 11 A0-wave signals acquired from the piezoelectric sensor array are labeled, $x_{{}_{i}}^{}(t),\;1\leq i\leq 11$, and the Green function from this point to the sensor array can be written as

(26) $$\bar{G}(R,r)=\left[X_{1}(\omega ),X_{2}(\omega ),\cdots X_{11}(\omega )\right]$$

where $X_{i}(\omega )$ is the Fourier transform of $x_{{}_{i}}^{}(t)$. In the experiment, the rectangular area from (−100 mm, −100 mm) to (100 mm, 100 mm) was scanned by a pulse laser in steps of 1 mm, which generated 40,401 green function vectors.

### 4.1. Single Damage Case

**Figure 19:**
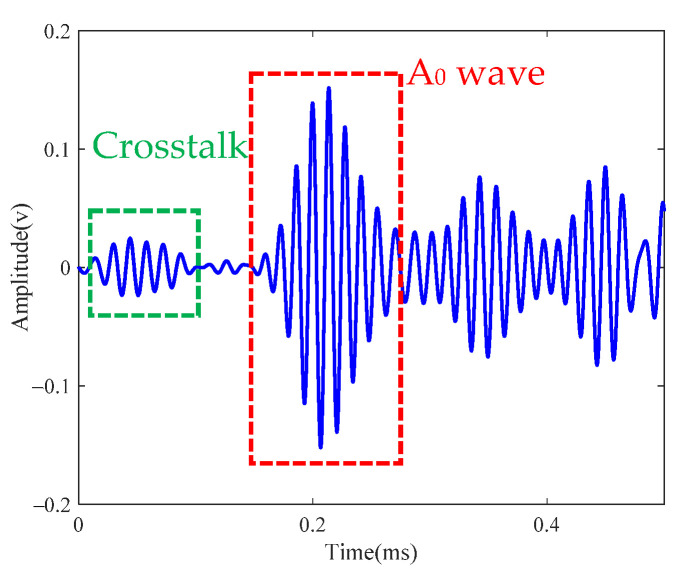
Scattered signal of single-damage case.

**Figure 20:**
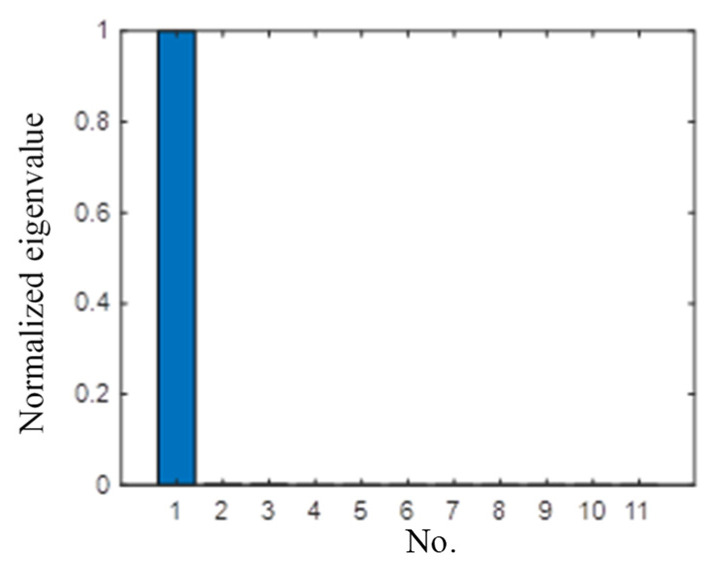
Normalize eigenvalue of single-damage case.

**Figure 21:**
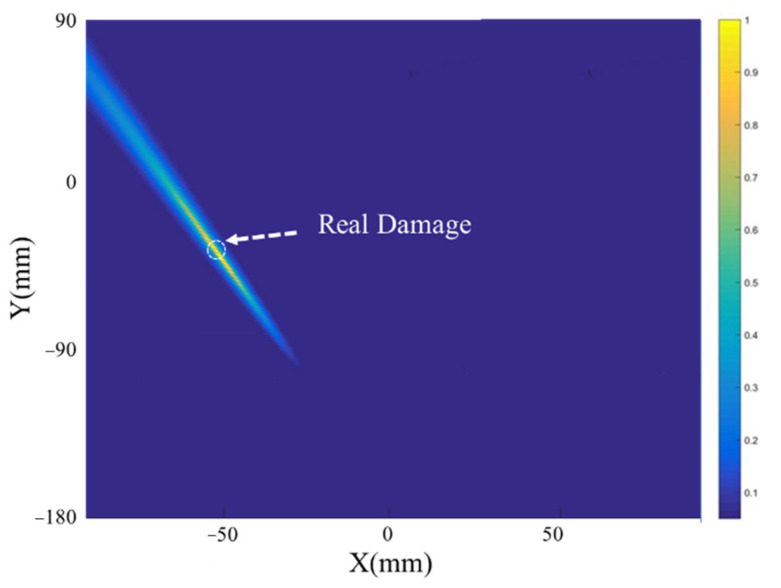
Damage image of single-damage case.

One drilled hole with a diameter of 4 mm was set at the location (−57 mm, −37 mm) in the coordinates of Figure 16b. Figure 19 shows the scattering signal transmitted and received by both sensors. The first wave is a crosstalk. The A0 wave is obvious and used to establish the transfer function matrix and perform the time-reverse and eigenvalue decomposition processes. Figure 20 shows the histogram of the normalized eigenvalue. It can be clearly seen in Figure 20 that there is only one nonzero eigenvalue that is related to the damage. The noise subspace and Green functions are substituted into Equation (22), and the damage image can be obtained as shown in Figure 21. It can be seen that the damage image gives the correct location of the real damage, while it also shows the typical feature of MUSIC whereby the angle resolution is obviously larger than the radial resolution.

### 4.2. Two-Damage Case

**Figure 22:**
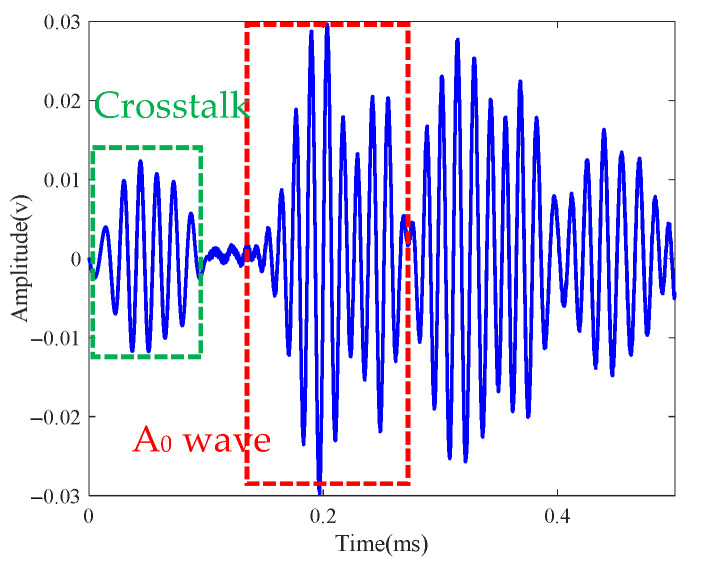
Scattered signal of two-damage case.

**Figure 23:**
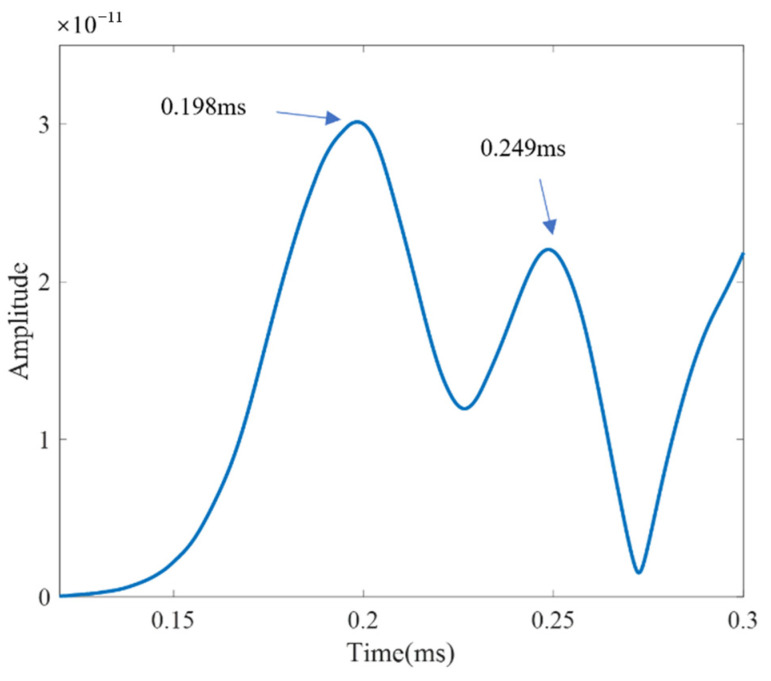
Time-dependent eigenvalue function for two-damage case.

**Figure 24:**
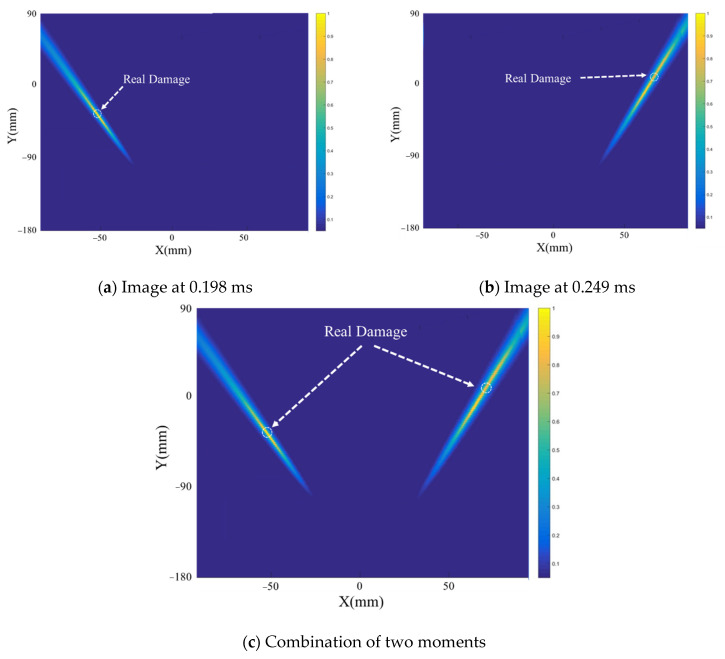
Damage image of two-damage case in the experiment.

Two drilled holes with diameters of 4 mm were set at the locations (−57 mm, −37 mm) and (70 mm, 8 mm). Figure 22 shows the scattered signal transmitted and received by both sensors. The first wave is a crosstalk. The A0 wave is longer than Figure 19 for the two-damage scattering, and this is used to establish the transfer function matrix and perform the time-reverse and eigenvalue decomposition processes. Local TR-MUSIC was used to analyze the A0 wave in Figure 22. Figure 23 gives the time-dependent eigenvalue function, in which there exist two peaks at the location of the scattering A0 wave. At 0.198 ms and 0.249 ms, damage images calculated by Equation (25) are given in Figure 24a,b, in which the locations of the pixel peaks show good consistency with the location of real damage. Combining Figure 24a–c gives the accurate locations of two real damages. However, due to the impact of noise, the TR-MUSIC damage image in the experiment has a worse radial accuracy than that in the simulation.

## 5. Conclusions

Damage imaging of plate-type structures based on Lamb waves can intuitively reveal the location and size of the damage, which has been a research topic of interest in the field of aircraft structural health monitoring. In this paper, an enhanced TR-MUSIC algorithm is proposed for multidamage detection in plate structures. The conclusions are as follows:

(1) The TR-MUSIC algorithm is constructed by the orthogonality of signal and noise subspaces, which are divided by the eigenvalue decomposition of the TR operation of the transfer matrix.
(2) An enhanced algorithm, the local TR-MUSIC algorithm, is proposed by using the moving time window to calculate the space spectrum of TR-MUSIC at different moments.
(3) The TR-MUSIC algorithm can effectively detect damage with a higher angle location accuracy than the radial location accuracy.
(4) The TR-MUSIC algorithm can break through the restriction of the Rayleigh criterion and realize the superresolution identification of multiple damage with distances smaller than a half-wavelength.
(5) By using the local TR-MUSIC algorithm conducted by a moving time window, the proposed algorithm can detect multiple damages, even though the number of identified damage can break through the limitation of the number of sensor array elements.
(6) The damage image in the experiment has a worse radial accuracy than that in the simulation due to the effect of noise.
(7) Some time-varying parameters, like temperature, load and so on, have a significant influence on Lamb waves. Although there are some focusing on eliminating the effect of these parameters, especially the effect of temperature, it is necessary to develop a suitable compensation method for the proposed TR-MUSIC algorithm in the future.

## Figures and Tables

**Table 1:** Damage cases.

Case	Damage Numbers	Damage Size (mm)	Damage Center (mm)
I	1	4 × 4 × 1	(20, 20)
II	3	4 × 4 × 1	(0, 100), (0, 0), (0, −50)

**Table 2:** Comparison of identified and real damage locations of three damage cases.

Damage No.	Real Damage Location	Identified Damage Location
1	(−50 mm, −50 mm)	None
2	(0 mm, 0 mm)	(0 mm, 1 mm)
3	(50 mm, 50 mm)	(100 mm, 60 mm)

**Table 3:** Eigenvalue of two damage with distance less than half-wavelength.

No.	Eigenvalue	No.	Eigenvalue	No.	Eigenvalue
1	2.15 × 10^−18^	5	9.10 × 10^−28^	9	1.06 × 10^−30^
2	1.70 × 10^−21^	6	9.71 × 10^−30^	10	4.37 × 10^−31^
3	1.09 × 10^−24^	7	3.39 × 10^−30^	11	2.69 × 10^−32^
4	9.41 × 10^−28^	8	1.83 × 10^−30^		

## References

[1] Qing X., Li W., Wang Y., Sun H. (2019). Piezoelectric Transducer-Based Structural Health Monitoring for Aircraft Applications. Sensors.

[2] Carboni M., Crivelli D. (2020). An acoustic emission based structural health monitoring approach to damage development in solid railway axles. Int. J. Fatigue.

[3] Saeedifar M., Zarouchas D. (2020). Damage characterization of laminated composites using acoustic emission: A review. Compos. Part B Eng..

[4] Sun H., Wang T., Lin D.W., Wang Y.S., Qing X.L. (2020). An Eddy Current-Based Structural Health Monitoring Technique for Tracking Bolt Cracking. Sensors.

[5] Sun H., Wang T., Liu Q., Qing X. (2018). A novel eddy current array sensing film for quantitatively monitoring hole-edge crack growth in bolted joints. Smart Mater. Struct..

[6] Sun H., Wang T., Liu Q., Wang Y., Qing X. (2021). A two-dimensional eddy current array–based sensing film for estimating failure modes and tracking damage growth of bolted joints. Struct. Heal. Monit..

[7] Neubeck R., Kexel C., Moll J. (2020). Matrix techniques for Lamb-wave damage imaging in metal plates. Smart Mater. Struct..

[8] Zhang H., Hua J., Gao F., Lin J. (2020). Efficient Lamb-wave based damage imaging using multiple sparse Bayesian learning in composite laminates. NDT E Int..

[9] Campos F.D., de Castro B.A., Budoya D.E., Baptista F.G., Ulson J.A.C., Andreoli A.L. (2019). Feature extraction approach insensitive to temperature variations for impedance-based structural health monitoring. IET Sci. Meas. Technol..

[10] Soman R., Singh S.K., Wandowski T., Malinowski P.M. (2020). Development of robust metric based on cumulative electrical power for electromechanical impedance based structural health monitoring. Smart Mater. Struct..

[11] Erdogan Y.S., Ada M. (2020). A computer-vision based vibration transducer scheme for structural health monitoring applications. Smart Mater. Struct..

[12] Fan G., Li J., Hao H. (2020). Vibration signal denoising for structural health monitoring by residual convolutional neural networks. Measurement.

[13] Roach D. (2009). Real time crack detection using mountable comparative vacuum monitoring sensors. Smart Struct. Syst..

[14] Mitra M., Gopalakrishnan S. (2016). Guided wave based structural health monitoring: A review. Smart Mater. Struct..

[15] Willberg C., Duczek S., Vivar-Perez J.M., Ahmad Z.A.B. (2015). Simulation Methods for Guided Wave-Based Structural Health Monitoring. Appl. Mech. Rev..

[16] Azuara G., Barrera E., Ruiz M., Bekas D. (2019). Damage Detection and Characterization in Composites Using a Geometric Modification of the RAPID Algorithm. IEEE Sens. J..

[17] Zhao J.J., Miao X.T., Li F.C., Li H.G. (2018). Probabilistic Diagnostic Algorithm-Based Damage Detection for Plates with Non-uniform Sections Using the Improved Weight Function. J. Vib. Eng. Technol..

[18] Bahador M.M., Zaimbashi A., Rahgozar R. (2020). Three-stage Lamb-wave-based damage localization algorithm in plate-like structures for structural health monitoring applications. Signal Process..

[19] Bao Q., Yuan S.F., Wang Y.W., Qiu L. (2019). Anisotropy compensated MUSIC algorithm based composite structure damage imaging method. Compos. Struct..

[20] Druet T., Recoquillay A., Chapuis B., Moulin E. (2019). Passive guided wave tomography for structural health monitoring. J. Acoust. Soc. Am..

[21] Druet T., Tastet J.-L., Chapuis B., Moulin E. (2019). Autocalibration method for guided wave tomography with undersampled data. Wave Motion.

[22] Nokhbatolfoghahai A., Navazi H.M., Groves R.M. (2019). Use of delay and sum for sparse reconstruction improvement for structural health monitoring. J. Intell. Mater. Syst. Struct..

[23] Ren Y.Q., Qiu L., Yuan S.F., Fang F. (2020). Gaussian mixture model and delay-and-sum based 4D imaging of damage in aircraft composite structures under time-varying conditions. Mech. Syst. Signal Process..

[24] Schmidt R. (1986). Multiple emitter location and signal parameter estimation. IEEE Trans. Antennas Propag..

[25] Engholm M., Stepinski T. (2011). Direction of arrival estimation of Lamb waves using circular arrays. Struct. Heal. Monit..

[26] Ambroziński Ł., Stepinski T., Uhl T. (2015). Efficient tool for designing 2D phased arrays in lamb waves imaging of isotropic structures. J. Intell. Mater. Syst. Struct..

[27] Zhang Z.H., Zhong Y.T., Xiang J.W. (2020). TAM and MUSIC Approach for Impact-Source Localization under Deformation Conditions. Sensors.

[28] Zhang Z.H., Zhong Y.T., Xiang J.W., Jiang Y.Y. (2020). Phase correction improved multiple signal classification for impact source localization under varying temperature conditions. Measurement.

[29] Zhang Z.H., Zhong Y.T., Xiang J.W., Jiang Y.Y., Wang Z.L. (2020). Research on the Performance and Improvement of Uniform Linear Sensors Array-Based Impact Localization Method Under Vibration Conditions. IEEE Sens. J..

[30] Yuan S., Zhong Y., Qiu L., Wang Z. (2015). Two-dimensional near-field multiple signal classification algorithm–based impact localization. J. Intell. Mater. Syst. Struct..

[31] Bao Q., Yuan S.F., Guo F.Y. (2020). A new synthesis aperture-MUSIC algorithm for damage diagnosis on complex aircraft structures. Mech. Syst. Signal Process..

[32] Devaney A.J., Marengo E.A., Gruber F.K. (2005). Time-reversal-based imaging and inverse scattering of multiply scattering point targets. J. Acoust. Soc. Am..

[33] Simonetti F. (2006). Localization of pointlike scatterers in solids with subwavelength resolution. Appl. Phys. Lett..

[34] Fan C., Pan M., Luo F. (2014). Ultrasonic broadband time-reversal with multiple signal classification imaging using full matrix capture. Insight-Non-Destr. Test. Cond. Monit..

[35] He J., Yuan F.-G. (2016). Lamb wave-based subwavelength damage imaging using the DORT-MUSIC technique in metallic plates. Struct. Heal. Monit..

